# An Experimental In Vivo Model to Characterize “Heavy Legs” Symptom in Topical Formulations

**DOI:** 10.1155/2009/547039

**Published:** 2010-03-11

**Authors:** Pedro Contreiras Pinto, Luís Monteiro Rodrigues

**Affiliations:** ^1^Experimental Dermatology Unit, Universidade Lusófona de Humanidades e Tecnologias, Campo Grande 376, 1749-024 Lisbon, Portugal; ^2^Experimental Physiology Laboratory, Faculdade de Farmácia, Universidade de Lisboa, Av. Prof. Gama Pinto, 1649-003 Lisbon, Portugal

## Abstract

The “Heavy legs” symptom is regarded as an early expression of chronic venous failure, estimated to affect 40% of the population in developing countries. A new methodology is proposed to approach the “tired or heavy legs” symptom. Seven females with this complaint applied a standard topical formulation during 28 days in one leg randomly chosen. Local blood flow records were obtained instantaneously and during postural change with a laser doppler flowmeter (LDF). High-frequency sonography and local morphometry were also obtained at Days 0, 14, and 28. When compared with D0, LDF values present a significant decrease of both basal and dynamical values after Day 14 and Day 28 suggesting that this effect may result from the formulation application, also involving the related massage. Centimetric measurements and sonographic analysis also supported those inferences. The proposed methodology can evaluate the dynamical changes of  “heavy legs” symptom and eventually be very useful to assess the related claim support.

## 1. Introduction

The “tired” or “heavy” legs sensation is one of the most frequently referred symptoms in early stages of vascular peripheral disease. This general designation includes a wide variety of pathologies, such as chronic venous insufficiency (CVI), which seems to affect up to 40% of the developed countries population [1, 2]. This helps to justify the relevant variety of medicinal (OTC) and health care products (cosmetics included), mostly topical cutaneous formulations, emerging in the last years to mitigate that condition. Depending on the regulatory space (EU, USA, or Japan) these formulations may even be considered as borderline products [3, 4]. In any case its efficacy and claim substantiation is far from being demonstrated, not only because the mechanisms explaining the symptom are still partially unknown but also because there is no directly related golden standard. 

A competent venous system comprehends a superficial and a deep circulation beds connected by perforating veins. The two circuits work together to store and return blood to the heart [5]. A series of valves and muscle pumps helps to keep the blood flowing, preventing the backflow. CVI appears when blood flow in the venous system is jeopardized, in most cases by valvular reflux, thrombotic obstruction of the valves, or both [5, 6]. In the progress of the pathology, there is an increase of the microvascular blood flow that causes the capillaries to become dilated and tortuous [5, 7]. This increases the hydrostatic forces that lead to edema, decreases oxygenation of the surrounding tissues, and evokes skin changes, often stasis dermatitis [5]. In the early stages of this evolution, subtle symptoms such as leg achiness, heavy legs, and leg tension sensations are often present [5–7]. Pathophysiology suggests that “tired” or “heavy” complaints should be related with the progressive dysfunction of peripheral vascular beds, involving structural, haemodynamical, and/or functional (local related autoregulation) capacities, although only a few papers addressed these issues [1, 2] in most cases by Laser Doppler Flowmetry (LDF), a widely known noninvasive technique. Apart from its recognized usefulness, LDF is known to suffer from several methodological limitations [8, 9] motivating other approaches. It is often associated to the topical application of vasoactive drugs [10, 11], to local heating [12–15], and to reactive hyperemia [16] by postural changes [17, 18] or by suprasystolic pressure cuff (or even other postural maneuvers), specially focusing the lower limb. These strategies help to reduce part of the methodological constrains and provide a dynamical-functional view over the vascular function [8, 16, 19, 20]. 

Concerning the referred topical formulations claiming to reduce the involved discomfort, one should note that “application” commonly involves a mechanical (massage) effect that should obviously be associated to any eventual reduction of the symptom expression, independently from any other therapeutical activity. But again, it would be necessary to properly design the experimental approach to quantitatively describe these eventual effects. 

This was the challenge reflected in the present paper, for example, to develop a sensitive methodology contributing to characterize the condition, eventually useful to substantiate the so-called “tired or heavy legs” improvement claims, for topical formulations. 

## 2. Material and Methods

### 2.1. Subjects

Seven female volunteers (mean age: 51.7 ± 8.6 y.o.) with healthy skin were selected from the database of the Experimental Dermatology Unit and included in the study. All the volunteers presented the “tired legs” complaint, confirmed by clinical evaluation, specially the presence of oedema, and a questionnaire. Inclusion criteria involved nonsmoking, no vasoactive medication or product, and restriction to drink coffee, tea, or any substance affecting microcirculation one day before lab evaluations. The presence of any pathology or symptom beside the “tired legs” was the main non-inclusion criteria. Volunteers were fully informed about the study objectives and gave their written consent. All procedures adopted were in full compliance with the Helsinki Declaration and its subsequent amendments. The project protocol was previously submitted and approved by the institution's Ethical Commission.

### 2.2. Formulation

An alkyl-acrylate crosspolymer gel formulation with no vasoactive substances was used. This polymer is one of the most popular component of “heavy (or tired) legs” market formulations being allegedly able to improve the sensation of discomfort implicit in the complaint, by a local massage effect [21]. 

### 2.3. Experimental Protocol

Volunteers were treated twice a day for 28 days, with 2 g of the formulation, applied in one randomly chosen lower limb (foot and leg), by gentle massage. The contralateral limb was used as a (negative) control. The study was therefore open to the volunteers and to the investigator. All observations and measurements were performed at day 0 (D0), at day 14 (D14), and at the end of the study (D28). All measurements were performed in a controlled room (Relative Humidity <50%; Temperature 21°C) following a 20-minutes stabilization.

The microcirculation blood flow, expressed in Blood Perfusion Units (BPUs) was assessed by the LDF system Periflux 5010 (Perimed, Stocholm, Sweden). Measurements were obtained **after** a steady local recording at rest, and **dynamically**, following a postural change like the sustained elevation of the limb from the seated position, by the same observer. This provocation induced a local (vascular) adaptative response eventually modifiable by the test product. 

High-frequency (16 MHz) sonography (Dermascan C—Cortex Technology, Denmark) was also used to complement the analysis. 

Foot and ankle (centimetric) perimeters were also registered.

### 2.4. Data Analysis

Statistically, the number of volunteers chosen is supported by adequate nonparametric approach. Descriptive statistics (MS Excel) and Wilcoxon Signed Ranks Test (SPSS 16.0) were applied, and a confidence level of 95% adopted.

## 3. Results

Results presented in Table 1 show a significant increase in the control leg blood flow, when compared with basal, after 14 and 28 days. In the treated leg, no statistical differences were found no matter the obvious increase in the mean values. It should be noted however that, during this period, climatic conditions did changed, with an increase in the ambient temperature after day 17th during the protocol execution. Results from the absolute variation during dynamical provocations are presented in Table 2. These show that in the treated leg there are no differences regarding D0, while in the control leg there is a variation increase, specially noted on Day 14.

**Figure 1:**
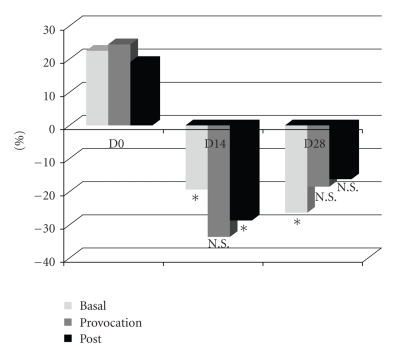
Differences in each evaluation period expressed as the variation against the control (*P < .05; NS: Nonsignificant: Comparison against D0).

In order to minimize the influence of intrinsic and extrinsic complicants (including the mentioned temperature changes), data were normalized by the quotient between the basal application value and the corresponding control in each evaluation day (Figure 1). 

Morfometric data were obtained by measuring the leg and ankle perimeters at D0, D14, and D28. Variations related to Day 0 and the corresponding statistics are summarized in Table 3. Although not significant, suggestive differences are seen in the nontreated limb (leg and ankle) at D14 and D28. 

**Figure 2:**
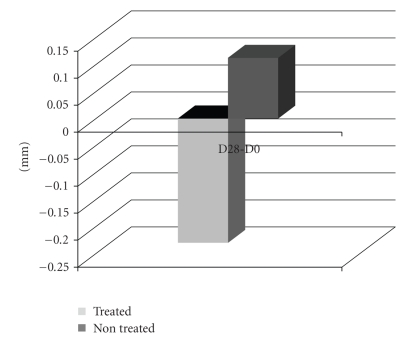
Variation related to D0 of the total cutaneous thickness for each evaluation area (mm).

**Figure 3:**
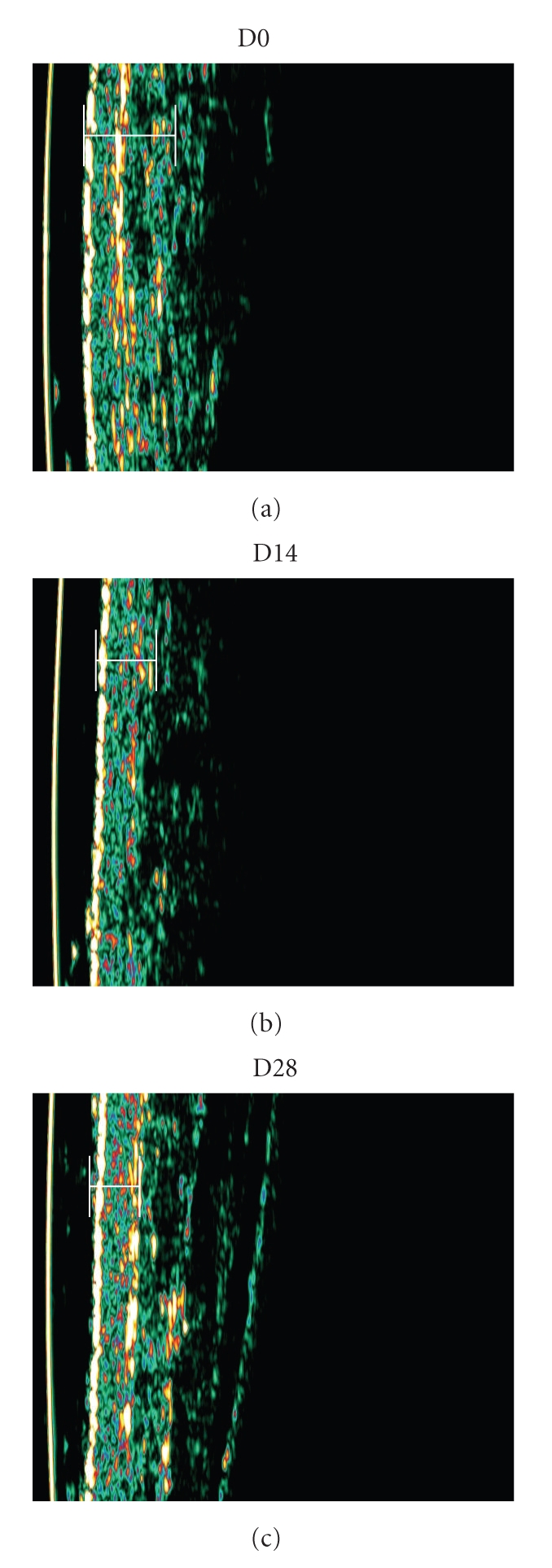
Illustrative examples of sonography images showing the apparent reduction in the full thickness area from D0 to D28 as a consequence of the treatment (vol#3).

Sonographic analysis of selected oedemacious areas was performed in a few volunteers (*n* = 3) to further explore and eventually illustrate the studied effects. Analysis took place at both internal and external faces of the ankle and included the total (cutaneous) thickness calculation. Results from D28 were very expressive (Figures 2 and 3) confirming a clear reduction of the oedema, when compared with D0, already detectable in D14. Additionally, in D28, volunteer's control leg presented an increase in the cutaneous thickness (0,11 ± 0,32 mm) while the treated leg has shown a reduction of −0.23 ± 0,21 mm at the same evaluation period.

## 4. Discussion

LDF is commonly used to assess local vascular conditions in a wide variety of pathologies from peripheral vascular disease [8] to diabetes [14, 15] or the Raynaud's syndrome [17, 18, 22]. LDF's dynamical assessment during limb postural changes proved to be very useful [8, 22], providing an interesting amount of information related with the local vascular function, especially regarding the veno-arterial reflex, although with different experimental approaches [8, 18, 22, 23]. In healthy subjects, a modification of posture from lying to standing involved an increase in the precapillary resistance of the foot's skin microcirculation, leading to an increase in local blood flow [20]. When the leg is lowered from supine, a decrease on the red cell flux was described [20]. Changing from supine to seated reportedly evoked a reduction in the lower limb blood volume [22]. More recently, an increase of the foot's blood flow by elevation of the leg (180° regarding the thigh) from seating was described [19]. Thus, these views provided a practical noninvasive, functional approach to study the impact of vasoactive substances or products on the peripheral perfusion conditions, offering new insights for comparative purposes.

When compared with D0, a significant reduction of basal LDF, in the treated area, 19% and 36% in D14 and D28, respectively, was noted, while provocation values decreased 33% and 18% in D14 and D28. Although significant differences in the provocation values could not be found, this clear tendency should be considered. This potential effect of minimizing the huge (microcirculatory) variations evoked by the ambient temperature rising was only present in the treated limb. As shown in Table 1, LDF values in the treated leg at D14 or D28, at rest, were similar to basal. A totally different condition was seen in the nontreated limb. Dynamical experiences corroborated those findings (Table 2) suggesting that the intervention, involving the formulation and the massage effect, determined some protective action that was maintained during the 28 days.

Morfometric results confirmed the minimizing effect of the intervention since, in the treated limb, the differences were clearly less evident.

Results from sonographic measurements at the selected oedemacious areas demonstrated the oedema and skin thickness reduction that can only be related with the formulation application, probably involving a local blood flow improvement. In the control leg the blood flow increase was pronounced, suggesting some involvement of the veno-arterial reflex which could induce or maintain the oedema and the high skin thickness [17]. 

## 5. Conclusion

No matter the present pilot-study approach and the apparent reduced number of volunteers, this methodology did prove to be able to characterize the vascular condition of each individual, allowing to discriminate the effects from a topical intervention on the peripheral vascular status of the lower limb. The proposed model may also help to look further to the vascular failure pathophysiology. 

## Figures and Tables

**Table 1:** Skin microcirculation blood flow obtained during single (basal) and dynamical (Provocation, Postprovocation) measurements at each evaluation day (*P < .05; NS: Nonsignificant: Comparison against D0).

BPU	Treated leg			Nontreated (control) leg		
	D0	D14	D28	D0	D14	D28
Basal	40.1 + 36.8	55.4 + 31.8^NS^	95.65 + 55.8^NS^	31.6 + 25.7	87.1 + 55.8*	148.9 + 126.7*
Provocation	64.9 + 48.5	66.5 + 37.3^NS^	111.4 + 56.7^NS^	58.3 + 42.3	107.4 + 62.3*	154.3 + 99.5*
Post provocation	26.7 + 29.6	36.3 + 20.1^NS^	105.7 + 75.9*	20.5 + 16.9	60.7 + 37.0*	131.6 + 95.6*

**Table 2:** Absolute variation means during the dynamical experiments.

BPU	Days		
	D0	D14	D28
Treated	21.0 + 31.6	22.0 + 27.9	18.5 + 17.8
Nontreated	25.3 + 35.5	34.5 + 37.9	21.6 + 24.5

**Table 3:** Variation related to D0 of the morfometric assessment for each evaluation area (cm) (*P < .05; NS: Non significant: Comparison against control variations).

cm	Leg	Ankle
Treated		
D14-D0	0.07 + 0.45^NS^	0.21 + 0.86^NS^
D28-D0	0.07 + 0.53^NS^	0.35 + 0.99^NS^
Non-treated		
D14-D0	0.29 + 0.64^NS^	0.14 + 0.75^NS^
D28-D0	0.14 + 0.56^NS^	0.21 + 0.76^NS^
